# Physicochemical Characteristics, Fatty Acid Profile, Alpha-Tocopherol Content, and Lipid Oxidation of Meat from Ewes Fed Different Levels of Distilled Myrtle Residues

**DOI:** 10.3390/molecules25214975

**Published:** 2020-10-27

**Authors:** Souha Tibaoui, Samir Smeti, Ines Essid, Juan Ramón Bertolín, Margalida Joy, Naziha Atti

**Affiliations:** 1 Laboratoire de Productions Animales et Fourragères, INRA-Tunisia, University of Carthage, rue Hédi Karray, Ariana 2049, Tunisia; souhatibaoui@gmail.com (S.T.); sam_fsb@live.fr (S.S.); 2 UR-UR PATIO (UR17AGR01), INAT, University of Carthage, 43 Avenue Charles Nicole, Tunis 1082, Tunisia; essidiness@gmail.com; 3 Centro de Investigación y Tecnología Agroalimentaria de Aragón (CITA), Instituto Agroalimentario de Aragón-IA2, CITA-Universidad de Zaragoza, Avda. Montañana 930, 50059 Zaragoza, Spain; jrbertolin@cita-aragon.es (J.R.B.); mjoy@cita-aragon.es (M.J.)

**Keywords:** food, sheep meat, color parameters, oxidative status, fatty acids, myrtle residues

## Abstract

The aim of this work was to study the sheep meat physicochemical traits as affected by distilled myrtle residues (MR) supplementation. For this, 27 culled ewes were divided into three groups receiving a ration composed by concentrate and hay for the Control group, concentrate and MR as a total substitute to hay for the Myrt-H group, or hay, less concentrate, and MR as a partial substitute to concentrate for the Myrt-C group. The meat chemical composition, pH, and color parameters were not affected by the MR intake. However, this animal’s dietary treatment resulted in higher meat polyphenol and α-tocopherol content for both MR groups (9.38 and 8.05 vs. 3.04 μg g^−1^ DM for Myrt-H, Myrt-C, and Control, respectively). In addition, since day 3 of meat storage, the lipid oxidation was improved by MR intake being lower for both MR groups than the Control (0.51 vs. 1.11 mg MDA/kg of meat). The total polyunsaturated fatty acid (PUFA) and saturated fatty acid (SFA) were similar among groups. However, the meat of Myrt-H had the highest C18:2n-6 and total PUFAn-6. In conclusion, the MR intake could be useful given it increases the meat content of vitamin E and improves its oxidative status without negative effects on the FA profile.

## 1. Introduction

As human food, the meat and meat products quality (color, fatty acid (FA) composition, lipid oxidation, etc.) depend on the feed of animals [1,2,3]. The consumer’s tendency of choice is oriented to foods rich in polyunsaturated FA (PUFA), with low saturated FA (SFA) content, since they are associated with increased LDL cholesterol [4]. On the other hand, meat color is the main factor influencing consumers’ purchasing choice, while flavor is judged during eating. Lipid oxidation is considered as one of the major deteriorating process of meat color and flavor; it is inevitable and irreversible that this phenomenon affected the meat traits during transformation, storage, and distribution, reducing the shelf life, leading to discoloration and off-flavor [5,6]. Apart from sensory quality deterioration, lipid oxidation causes oxidative reactions in the meat through the degradation of PUFA, which generate residual products, such as malondialdehyde (MDA) and lipid-derived volatiles [7]. For this, the use of synthetic antioxidant was widely practiced as an ingredient in animal diet or as an additive in meat products. However, they may be associated to anti-nutritional effects or toxicological consequence, so the use of such substances became rejected by consumers [8]. Smart animal nutrition strategies are now using natural antioxidants in order to satisfy consumer’s demands for a healthier product [9,10]. Hence, the incorporation of aromatic and medicinal plants or their extract in animal’s diet may be considered as an alternative method to inhibit or delay meat oxidation. Given their richness in phenolic compounds, these plants could be a major determinant of the antioxidant potentials of meat [11]. The antioxidant power of plant extracts or essential oils has been reported in various meats such as pork [12] and lamb meat [1,11,13]. The distillation industry of aromatic and medicinal plants generates substantial quantities of phenolic-rich by-products, which could be valuable natural sources of antioxidants. Some of these by-products, such as rosemary and thyme distillate leave, have been the subject of several investigations and have proven to be effective sources of phenolic antioxidants [10,14,15]. Myrtle (*Myrtus communis* L.) is an aromatic plant shrub distributed throughout the Mediterranean area that is widely used in folk medicinal practices as an antimicrobial and antioxidant. Several authors investigated the use of myrtle essential oil in the poultry diet [16,17]. Distilled myrtle residues (MR) are discarded after the extraction of essential oils; they could be an interesting alternative feed for animals given their protein and energy content and their richness in bioactive compounds [18], especially in regions suffering from insufficient feeding resources. On the other hand, culled ewes offered great quantities of meat; however, this meat is usually tough and fibrous in texture [19], it accumulates more branched-chain fatty acids [20], and it is more susceptible to oxidation; thus, it became less preferred by the consumers [19].

Many researchers studied the possibilities of using fodder shrubs and agro-industrial by-products as alternative feed resources [21] or as a natural antioxidant [22]. However, references on the effect of MR in ruminants feed on meat properties are nonexistent. Therefore, the aim of this study was to evaluate the physicochemical quality and oxidative stability for the meat of culled ewes fed hay and concentrate as control compared to that fed MR as a total substitute to hay (Myrt-H) or partial substitute to concentrate (Myrt-C).

## 2. Results

### 2.1. Meat Chemical Composition and α-Tocopherol Content

No differences attributable to the dietary treatment were observed for meat proximate composition or cholesterol amount. The intramuscular fat content was about 20% DM, while protein content averaged 71% DM in all groups. However, polyphenols and α-tocopherol contents were higher (*p* < 0.01) for the meat of both MR groups than the control (Table 1).

### 2.2. Lipid Oxidation and Color Stability of Meat during Refrigerated Storage

Thiobarbituric acid reactive substances (TBARS) values were significantly affected by diet and storage time (Table 2). At day 0, no differences were found between dietary treatments. The meat of both MR groups was kept constant with low values that ranged between 0.35 and 0.57 mg MDA/kg until day 3 to reach 1.2 and 1.06 mg MDA/kg at day 9 of storage for Myrt-H and Myrt-C, respectively. However, the meat of C group had values above 1 mg MDA/kg since day 3 and reached values of more than 1.5 mg MDA/kg at the end of the storage time.

Neither MR supplementation nor the interactions between storage time and diet have a significant effect (*p* > 0.05) on meat color parameters (Table 2). Hence, all parameters had similar values for all groups. However, the color parameters significantly decreased during the storage time. During storage, ΔE * ranged between 3.81 and 5.23, which was recorded for Myrt-H and C groups, respectively.

### 2.3. Fatty Acids Profile

The lipid meat’s individual FA, the class of FA, and ratios are presented in Table 3 and Table 4. For all groups, the SFA and MUFA represent around 89% of the quantified FA, where oleic acid (C18:1-9) was the most abundant with a mean percentage of 31.2%, followed by palmitic acid (C16:0) and stearic acid (C18:0). The level of branched fatty acid (BCFA) was similar among all groups. No significant difference was recorded for the sum of SFA, MUFA, and PUFA contents among treatments (Table 4). The concentration of total detected conjugated linoleic acid (CLA) isomers was low for all groups. The linoleic fatty acid (C18:2 n-6) content was significantly higher for the Myrt-H group (Table 3). For total trans-18:1 and individual isomers content, no differences were observed among groups, except for trans-11 C18:1, vaccenic acid (VA) where the Myrt-H group presented the highest concentration (*p* < 0.05).

The Myrt-H group had the lowest values of C18:1-10t/C18:1-11t ratio (*p* < 0.05). The meat of the Myrt-H group presented a higher (*p* < 0.05) n-6/n-3 PUFA ratio (Table 4), and it tends to have the highest level of PUFA n-6 among all groups. The levels of minor n-6 PUFA, C20:3 n-6, and C20:4 n-6 ARA were significantly higher for Myrt-H. The concentration of long-chain n-3 PUFA including 20:5n-3 (EPA), 22:5n-3 (DPA), and 22:6n-3 (DHA) was not modified due to MR supplementation. The desirable fatty acids (DFA) content tends to be higher (*p* = 0.08) for the C and Myrt-H groups, with 65.62% and 64.52%, respectively (Table 4).

## 3. Discussion

The similarity in meat chemical composition among groups may be due to the similarity of the animal’s slaughter body weight [23]. In a recent study, Yagoubi et al. [15] have reported higher protein content and less intramuscular fat content in lamb’s meat when animals were fed rosemary distillation residues. Hence, adding linseed oil and natural Vitamin E into ewes’ diet has proven to only affect lamb meat’s fat and ash content [24]. The higher antioxidant ability of meat during storage for MR groups may be explained by the higher total phenolic content (TPC) and DPPH radical scavenging ability in both MR pellets (M-Hay and M-Conc) compared to hay and concentrate (Table 5). Several authors have reported the effect of animal’s diet rich in TPC on their meat’s polyphenols and tocopherol’s content. Moñino et al. [1] showed a positive transfer of polyphenols to lamb meat when ewes were fed rosemary by-product. Similar results were found with lambs fed rosemary residues rich in polyphenols [15]. Despite the presence of other tocopherols, α-tocopherol is the principal component of the vitamin E activity. It was shown that α-tocopherol can be used as an antioxidant in animal’s diet, since it has the capacity to delay meat oxidation. In fact, α-tocopherols are not degraded in the rumen but instead are deposited in muscle cell membranes where their antioxidant actions are more effective [25,26]. This finding is in agreement with Gómez-Cortés et al. [26], who found a similar result with lamb meat when ewes were fed grape pomace. Hence, several authors reported the effect of dietary supplementation on muscle’s α-tocopherol content. Ripoll et al. [27] found an increase in muscle’s α-tocopherol content when lambs received α-tocopherol supply; the same tendency was observed with meat produced on pasture and fresh forage, which are rich in α-tocopherol [28]. In reverse, the linseed oil supply did not affect the vitamin E levels in ewes meat [24]. The meat lipid oxidation was decreased by feeding MR to ewes. The decrease of TBARS values during storage in both MR groups may be explained by the presence of α-tocopherol, which is known for its antioxidant ability [25], explaining the high correlation between TBARS and α-tocopherol values (r^2^ = 0.95). These results are in concordance with the polyphenols and α-tocopherols content previously reported for the current study. Polyphenols are also proven to have strong antioxidant effects against reactive oxygen species and free radical attacks in biological systems and in foods [29]. Several authors mentioned that polyphenols have the ability to prevent muscle tissues from oxidation [26]. Similar results were found by other researchers on the meat lipid oxidation delay by rosemary residues supply to lamb [15,30]. Escalante et al. [31] explained this result by the ability of rosemary extract to chelate metal ions, which lead to a decrease in the formation rate of activated oxygen. In this context, Kerry et al. [32] confirmed that the dietary antioxidants incorporated within cell membranes are more efficient than those added post mortem to preserve meat products from oxidative damage and to extend their shelf life. Hence, Moñino et al. [1] showed that phenolic compounds can be transferred to lamb meat from ewe fed rosemary distillate leaves. Phenolic compounds might have an indirect antioxidant effect, since they can chelate pro-oxidant metals and reduce the production of peroxides [33]. Color loss during storage may be attributed to the oxidation process in the presence of oxygen. In fact, the lightness is mostly associated with muscle and protein structures, which influence the water holding capacity [34]. The oxidation can cause an increase in cell membrane permeability and thus a decrease in the water holding capacity of myofibrils, and, as a consequence, more juice loss from the meat and an increase in L * [35]. At the end of the storage, the meat of the Myrt-H and control groups had the highest value of redness (a *) and yellowness (b *), respectively. Our results are in agreement with those of Realini et al. [36], who showed no feeding effect on meat color parameters. The effects of animal diet on color have been largely described in fresh meat [13]. In addition, it was shown that meat’s color stability is associated to oxidation [37], and that rosemary residues, with high antioxidant power, extended the sausages’ color stability during storage [10]. Color differences (∆E *) are described in the National Bureau of Standards Unit (NBS unit) and state that there are small differences between colors, which are detectable by humans: 0–0.5 trace, 0.5–1.5 slight, 1.5–3.0 noticeable, 3.0–6.0 appreciable differences, 6.0> obvious difference. Based on those standards, in the current study, color changes were perceptible, especially with the C group, where (∆E *) was around 5.32. These results confirm that storage time did have a significant effect on meat’s color, which was more noticeable with C group samples.

Lipid auto-oxidation products were found to increase oxy-myoglobin oxidation [38]; thus, the incorporation of dietary plant extract may inhibit color degradation. Studying the incorporation of grape pomace and linseed oil into ewes’ diet, Gómez-Cortés et al. [26] and Gallardo et al. [24] reported that polyphenols supply can inhibit color deterioration during storage due to their ability to reduce myoglobin oxidation.

The results on the prevalence of oleic, stearic, and palmitic FA in sheep meat is in concordance with the literature [11,36,39]. From a nutritional point of view, the MR supply had a positive effect since palmitic acid (C16:0) and myristic acid (C14:0) present lower concentrations in MR groups. In fact, SFA with lengths from 12 to 16 carbons can raise cholesterol concentration and increase inflammation and risks for Cardiovascular disease (CVD) in human [4]. The similarity in BCFA among groups may be due to the fact that all diet had similar carbohydrate contents; in fact, BCFA are synthetized from the propionate originated in the rumen by the fermentation of dietary carbohydrate [40]. Similar results were found on meat from lambs supplied by linseed and quercetin [39]. However, several authors found that animal’s diet affected the BCFA content in ruminant products. The meat of lambs fed concentrate had a higher BCFA concentration than the meat of lambs fed on pasture [41]. These FA are responsible for the distinctive sheepy flavor and odor in meat, which are considered important factors for consumers [40]. Hence, an undesirable flavor of cooked meat may be attributed to their medium-chain fatty acids content, especially, 4-methyloctanoic and 4-methylnonanoic acids [42]; in the current study, these compounds have not been identified. The low concentration of total CLA detected in the meat from all groups could be due to the dietary PUFA conversion in the rumen [24]. Myrt-H had the lowest values of C18:1-10t/C18:1-11t ratio due to its higher vaccenic acid content (C18:1 trans11). Gómez-Cortés et al. [26] explained similar results by the absence of changes on the ruminal biohydrogenation pathway, which leads to an accumulation of vaccenic acid in the meat. Human *trans* FA intake should be as low as possible, considering their negative potential effect on human health [43]. Thus, the MR diet improved meat’s VA content, which is known for their health benefits. An improvement was recorded in lamb meat’s *trans* FA content when the ewe diet was supplemented with linseed oil and vitamin E [24]. The higher n-6/n-3 PUFA ratio and PUFA n-6 content may be associated to the n-6/n-3 PUFA dietary ratio and PUFA n-6 content in feedstuffs [44]. Diets with thyme leaves, rosemary residues, or rosemary essential oil resulted in a decrease in the n-6/n-3 ratio in lamb meat [11,14,21]. Meanwhile, Resconi et al. [45] found no significant difference in n-6/n-3 ratio in meat from suckling lambs reared by ewes fed grape pomace and grape seed. On the other hand, Myrt-H had higher levels of Vitamin E; in fact, Enser et al. [46] reported that vitamin E concentration and n-6 levels are associated, which suggest that higher levels of antioxidant inhibited PUFA loss. The diets used in this study did not affect the long chain n-3 PUFA content. Guerrero et al. [47] reported a lack of effect of linseed administration, into cull ewes diet, on EPA and DHA concentrations. They explained this result by the competition between α-linolenic acid and the precursor for DHA for the activity of the Δ6 desaturase enzyme. However, long-chain n-6 PUFA was significantly affected by the diet, where Myrt-H had the highest C20:3 n-6 and C20:4 n-6 ARA content (*p* < 0.05). The thrombogenic index (TI) and atherogenic index (AI) were not affected by the dietary treatment. The TI averaged 1.6 for all groups, while AI values varied between 0.69 for the C group and 0.76 for the Myrt-C group. Similar AI values were also found by Nieto et al. [14] in lamb’s meat when ewes were fed thyme leaves in the diet. Myrt-H samples also tend to have the highest DFA content. This result may be explained by the higher C18:0 and PUFA content. PUFA are the most sensitive to oxidation due to the presence of double bonds in the hydrocarbon chain [48]. Thus, PUFA are more exposed to oxidative reactions, since they constitute a part of the phospholipids located in cellular membranes [49]. Dietary supplementation, which has beneficial effects on meat’s antioxidant stability, may influence the PUFA content. Thus, due to its high content in polyphenols, the Myrt-H diet maintained the unsaturated FA level in cell membranes, particularly C20:3 n-6, C20:4 n-6 ARA, and vaccenic acid content (C18:1 trans11) [50]. Similarly, Recsan et al. [51] reported that dietary supplementation with aromatic plant essential oil did protect PUFA against oxidation in cell membrane.

## 4. Material and Methods

### 4.1. Diets, Animals, Experimental Design, and Slaughter Procedure

All the procedures employed in this study (transport and slaughtering) meet ethical guidelines and adhere to Tunisian legal requirements in accordance with Law no. 2005-95 of 18 October 2005 (Chapter II; Section 1 and Section 2 relative to the slaughter of animals).

The MR were collected from a distillation unit in the Northwest of Tunisia (Nefza) and then air-dried to ensure complete dehydration. Then, dried MR were ground and mixed with other ingredients to make two types of pellets. The M-Hay pellets were composed from 87% MR and 13% bran to have a feed similar to oat hay. The M-Conc pellets contain 30% MR, 12% soy, and 58% barley; it is equivalent to a concentrate. Twenty-seven Barbarine culled ewes (35 kg of body weight, BW) were divided into three homogenous groups of 9 each and were reared in individual pens. Sheep in the control group (C) received 750 g of concentrate and 500 g of hay. The Myrt-H group received 750 g of concentrate and 500 g of M-Hay as a whole substitute to hay. The Myrt-C group received 500 g of oat-hay, 350 g of concentrate, and 400 g of M-Conc in partial substitution to concentrate. The feed’s antioxidant potential and nutritional composition are shown in Table 5. All ewes were slaughtered at similar BW (41.5 kg) at the end of the fattening period, which lasted 90 days. After cooling at 4 °C during 24 h, carcasses were divided into two halves, and the *Longissimus thoracis* and *lumborum (LTL)* muscle was extracted from the left side for meat analysis.

### 4.2. Meat Samples Preparation and Measurements

Samples of *Longissimus thoracis and lumborum (LTL*) muscle were stored at −20 °C for Vitamin E and FA analysis. A section of LTL muscle was cut into four 2.5 cm thick slices, wrapped with an oxygen-permeable polyvinylchloride film, randomly assigned to four 4 trays (0, 3, 6, or 9 d of storage), and kept in darkness at 4 °C. Immediately after color measurement, the samples were packed and frozen (−20 °C) until TBARS analysis. In another section, the initial pH and ultimate pH (1 h and 24 h after slaughter, respectively) were measured with a penetrating electrode connected to a pH meter (HI 99163; Hanna Instruments, Sălaj, Romania) and calibrated with two buffers (7.00 and 4.01) at 25 °C.

To evaluate color, a Minolta spectrophotometer (CM-2006 d; Konica Minolta Holdings, Inc., Osaka, Japan) was used directly on the muscle surface with a measured area of 8 mm, standard illuminant D65, and an observer angle of 10°. The lightness (L *), redness (a *), and yellowness (b *) were recorded. Hence, (ΔE *) was calculated in order to estimate the color change during storage time, using the following equations: ΔE * = [(L_0_ − L_t_)^2^ + (a_0_ − a_t_)^2^ + (b_0_ − b_t_^2^)]^1/2^, where 0 is the value at day 0 and t is the value at day 9 [52]. Results were expressed according to the CIE L * a * b * system (CIE, 1976) as the average of three measurements performed on a cut surface area. For chemical composition, samples of meat were lyophilized to obtain dry matter (DM); then, samples were ground (1 mm screen). On homogenized ground samples, ash was determined by incineration at 600 °C for 8 h, nitrogen was determined by the Kejeldahl method, and then, the crude protein content was calculated as N × 6.2 [53]; the fat was extracted with petroleum ether, which was measured using AOCS Standard Procedure Am 5-04.

### 4.3. Lipid Oxidation Analyses (TBARS) in Meat

Meat lipid oxidation was assessed by the TBARS (thiobarbituric acid reactive substances) assay as determined by Botsoglou et al. [54] with modifications. First, 20 mL of 10% trichloro acetic acid were added to 10 g of minced meat, and homogenization was carried out using an Ultra-Turrax (T25, IKA-Labortechnik, Staufen, Germany) for 10 s at 13,500× *g* rpm. After homogenization, 2 mL of the supernatant obtained was transferred to a test tube and mixed with 2 mL of thiobarbituric acid solution. Test tubes were incubated at 97 °C in a water bath for 15 min to develop a pink absorbance, and the absorbance of the sample was recorded against the appropriate blank at 532 nm by a spectrophotometer (Shimadzu, Tokyo, Japan). A calibration curve was prepared using 1,1,3,3-tetramethoxypropane; and TBARS values are expressed as mg malondialdehyde (MDA) equivalents/kg sample.

### 4.4. Total Phenolic Content (TPC) and Vitamin E Analysis

The TPC of meat was performed, in triplicate, according to the method of Vázquez et al. [55] with modifications. To 1 g of ground meat, 9 mL of milli-Q water and 10 mL of aqueous solution of methanol (50/50; *v*/*v*) were added and mixed for 5 min. To the obtained mix, 500 μL of Carrez I solution were added and dissolved while vortexing for 1 min; then, 500 μL of Carrez II solution were added and dissolved while vortexing for 1 min, and finally, 5 mL of acetonitrile were added and dissolved. The test tubes were left to stand for 25 min and then centrifuged at 4000× *g* rpm for 15 min at 4 °C. The supernatant was filtered through a 0.22 μm PTFE filter in a 15 mL tube, and the extract obtained was used to determine TPC. The TPC in the liquid extract was estimated using the Folin-Ciocalteu method. To 15 µL of extract, 147 µL of water milli-Q, 13 µL of Folin-Ciocalteu reagent, and 7% Na_2_CO_3_ were added. Samples were held to stand for 1.5 h in the dark. The absorbance of samples was measured with a spectrophotometer (Thermo Electron Corporation, Spain) at 750 nm, and the results were expressed as μg gallic acid equivalents (GAE)/g dried sample. The ABTS assay was determined following the method of Saura-Calixto and Goñi [56] with some modifications. The vitamin E analysis was performed according to the method of Prates et al. [57]. For saponification, 0.2 g of ascorbic acid and 3 mL of saponification solution were added to 0.2 g of lyophilized meat; the mix was placed in glass tube of 25 mL. The saponification solution was made by mixing 10% *w*/*v* of KOH with 50% EtOH and 50% of distilled water. The tubes were vortexed under nitrogen atmosphere, in order to eliminate the air, and then left overnight in an orbital shaker at (600× *g* rpm). In the tubes, 5 mL of hexane-ethyl acetate (9:1 *v*/*v*) and 5 μg/mL of 2,6-di-tert-butyl-4-methylphenol (BHT) were added. The mixture was shaken with a vortex for 5 s, followed by the orbital shaker at maximum speed for 10 min and centrifugation for 5 min at 3500× *g* rpm and 10 °C in order to separate different phases. An aliquot of the upper layer was transferred into a glass tube of 10 mL and then evaporated in a rotational vacuum concentrator (Christ RVC2-25) for 45 min at 40 °C. The residue obtained was dissolved in 1 mL of mobile phase acetonitrile/methanol/dichlorometane (75:15:10, *v:v:v*). The mixture was vortexed and shaken in an orbital shaker (600× *g* rpm) for 10 min, and the aliquot of the n-hexane layer was filtered through a 13 mm × 0.20 μm PTFE filter into an amber screw-cap vial for UPHLC. The chromatographic system used was an Acquity UPLC H-Class liquid chromatograph composed by a fluorescence detector (Waters 2475 Multi Fluorescence Detector) and a column Acquity UPLC HSS T3 column 2.1 mm × 15 mm × 1.8 μm. Tocopherols and cholesterol were detected at 295 and 220 nm, respectively.

Total phenolic content and antioxidant activity analysis in feedstuff samples was previously described in Tibaoui et al. [58].

### 4.5. Fatty Acid Profile

Meat’s fatty acid methyl esters (FAME) were assessed according to the method of Lee et al. [59], with modifications. For this, 0.2 g of lyophilized and minced meat samples were mixed with 1 mL of internal standard (Methyl tricosanoate, C23:0), 2 mL of heptane, and 4 mL of NaOH/CH3CH (0.5 M). The mixture was homogenized with vortex (30 s) and then heated for 20 min at 50 °C. After cooling, 4 mL of acetyl chloride was added CH_3_OH (1/10 *v*/*v*), and the mixture was homogenized and heated again for 60 min at 50 °C. After cooling at ambient temperature for a few minutes, 2 mL of water milli-Q was added; then, the mixture was homogenized and centrifuged for 5 min, 3500× *g* rpm at 10 °C. The upper layer was extracted and deposited into 5 mL tubes where anhydrous Na_2_SO_4_ was introduced for dehydration. Then, the mixture was shaken and centrifuged for 5 min, 3500× *g* rpm at 10 °C; 1 mL of the supernatant was transferred into a screw-cap glass vial for gas chromatography. FAME analyses were carried out by gas chromatography (GC-FID Bruker gas chromatograph) equipped with a capillary column (BR-2560). The identification of FAME was aided with the use of several reference methyl esters (GLC-532, GLC-401, GLC-642, GLC-643, GLC-538 and GLC-463) and quantified using the internal standard (C23:0 Methyl-tricosanoate). Saturated (SFA), monounsaturated (MUFA), and polyunsaturated fatty acids (PUFA) were calculated and reported as % FAMEs. The desirable fatty acids were calculated as DFA = ∑MUFA + ∑PUFA + C18:0. The saturation index (SI) (1), atherogenic (AI) (2) and thrombogenic (TI) (3) indices were calculated according to Ulbricht and Southgate [60]:

(1) $$SI=\frac{\left(C14:0\;+\;C16:0\;+\;C18:0\right)}{\sum MUFA+\;PUFA}$$

(2) $$AI=\;\frac{\;C_{12:0}+\;\left(4\;x\;C_{14:0}\right)+C_{16:0}}{\sum MUFA+\;\sum n6\;+\;\sum n3}$$

(3) $$TI=\;\frac{\;C_{14:0}+\;C_{16:0}+C_{18:0}}{0.5\;\sum MUFA+\;0.5\;\sum n6\;+\;3\sum n3+\frac{\sum n3}{\sum n6}}$$

### 4.6. Statistical Analysis

The effect of dietary treatment on the meat’s FA profile, chemical composition, and antioxidant activities measured were assessed by one-way ANOVA, using the General Linear Model (GLM) procedure of SAS (2004) with Student’s multiple range test, and the statistical significance was defined at *p* < 0.05. For pH, color, and TBARS measurements during storage, a Linear-Mixed model analysis was conducted, with diet, storage time, and the interaction between them as the variable factors. Mean squared errors (MSE) were determined for all analyses.

## 5. Conclusions

Meat color parameters were not affected by ewes’ intake of myrtle residues. However, healthy FAs, including long-chain PUFAs such as C20:3 n-6 and C20:4 n-6 ARA, vaccenic acid, and DFA were higher in the Myrt-H goup. In addition, these residues lead to higher meat α-tocopherol and TPC content and better lipid oxidation by lowering the TBARS values. It seems that polyphenols have the ability to transfer into muscle from the diet, which could contribute to enhancing the meat’s antioxidant stability and improve its fatty acid profile. Therefore, according to the results obtained, the substitution of conventional feedstuff with a dose of 87% MR in ewes’ diet could be an interesting alternative to improve the meat quality of culled ewes, being an inexpensive source of α- tocopherol and TPC, protecting PUFA against oxidation, reducing the level of lipid oxidation, and improving color stability during storage.

## Figures and Tables

**Table 1:** Antioxidant activity and chemical composition (% DM) of meat from ewes fed myrtle residues.

	C	Myrt-H	Myrt-C	*p*	SEM
**Polyphenols**	53.02 ^c^	72.07 ^a^	68.74 ^b^	0.04	5.87
**ABTS**	0.90	0.89	0.86	0.74	0.01
**α-tocopherol** (μg/g DM)	3.04 ^b^	9.38 ^a^	8.05 ^a^	<0.01	1.93
**γ-tocopherol** (μg/g DM)	0.27 ^a^	0.16 ^b^	0.19 ^b^	0.01	0.03
**Cholesterol** (mg/g)	1.96	2.05	2.05	0.86	0.03
**Dry matter**	28.19	27.38	27.68	0.72	0.23
**Ash** (% DM)	4.60	4.59	4.60	0.10	0.03
**Protein** (% DM)	71.50	71.60	71.30	0.58	0.09
**Fat** (% DM)	23.90	23.81	24.1	0.48	0.08

C: Control group receiving oat hay and concentrate; Myrt-H: group receiving pellets containing 87% myrtle residues (MR); Myrt-C: group receiving pellets containing 30% MR; a. b. c: different letters within the same row (different diet) differ significantly (*p* < 0.05).

**Table 2:** Color parameters and lipid oxidation evolution of meat from ewes fed myrtle residues.

	**Diet**	**Day 0**	**Day 3**	**Day 6**	**Day 9**	***p* Diet**	***p* Time**	*p* Diet * **Time**	**SEM**
	**C**	0.42 ^a^	1.11 ^by^	1.58 ^cy^	1.98 ^cy^				
**TBARS**	**Myrt-H**	0.35 ^a^	0.57 ^ax^	0.78 ^ax^	1.2 ^bx^	0.01	0.01	0.14	0.20
	**Myrt-C**	0.43 ^a^	0.46 ^ax^	0.62 ^ax^	1.06 ^bx^				
L *	**C**	35.49	34.29	33.47	32.71 ^y^	0.26	0.05	0.10	1.32
	**Myrt-H**	33.32 ^a^	32.67 ^a^	31.21 ^b^	30.45 ^bz^				
	**Myrt-C**	37.77	37.15	36.3	34.8 ^x^				
a *	**C**	17.24 ^a^	16.45 ^a^	15.96 ^a^	12.96 ^by^	0.99	0.05	0.50	0.04
	**Myrt-H**	16.4 ^a^	15.9 ^a^	15.38 ^a^	14.37 ^bx^				
	**Myrt-C**	16.75 ^a^	16.27 ^a^	15.26 ^a^	14.02 ^bx^				
b *	**C**	6.74 ^a^	6.55 ^a^	5.94 ^a^	5.68 ^ax^				0.29
	**Myrt-H**	6.28 ^a^	5.87 ^a^	5.45 ^ax^	4.8 ^by^	0.77	0.05	0.60	
	**Myrt-C**	5.92 ^a^	5.53 ^a^	4.93 ^abx^	4.55 ^by^				
**Color change**									
	**C**	5.23							
ΔE *	**Myrt-H**	3.81							
	**Myrt-C**	4.26							

C: Control group receiving oat hay and concentrate; Myrt-H: group receiving pellets containing 87% myrtle residues (MR); Myrt-C: group receiving pellets containing 30% MR; L * (lightness); a * (redness); b * (yellowness); a. b. c: different letters within the same row (different storage days) differ significantly (*p* < 0.05); x. y. z: different letters within same columns (different diets) differ significantly (*p* < 0.05); ΔE *: total color change for each group during storage.

**Table 3:** Fatty acid profile (% of total fatty acid methyl esters (FAME)) of meat from ewes fed myrtle residues.

Item	C	Myrt-H	Myrt-C	*p*	SEM
**SFA**					
C10:0	0.35	0.43	0.45	0.45	0.03
C12:0	0.10	0.11	0.11	0.66	0.003
C14:0	2.11	2.30	2.19	0.66	0.06
C15:0	0.42	0.42	0.34	0.17	0.03
C16:0	24.51	24.99	26.25	0.13	0.52
C17:0	1.28	1.28	1.07	0.19	0.07
C18:0	15.69	16.52	15.81	0.56	0.26
C19:0	0.12	0.10	0.08	0.41	0.01
C20:0	0.10	0.09	0.11	0.082	0.01
C22:0	0.04	0.04	0.05	0.82	0.002
**MUFA**					
C14:1	0.07	0.08	0.08	0.80	0.05
C16:1 *cis*9	1.31	1.34	1.49	0.48	0.003
C16:1 *trans*9	0.02	0.01	0.02	0.31	0.002
C16:1 *trans*10	0.06	0.05	0.06	0.90	0.02
C17:1 *cis*9	0.16	0.12	0.11	0.56	0.74
C18:1 *cis*9	32.56	30.00	31.08	0.43	0.03
C18:1 *cis*11	1.30	1.37	1.28	0.54	0.005
C18:1 *cis*12	0.20	0.22	0.21	0.86	0.0014
C18:1 *cis*13	0.13	0.13	0.12	0.97	0.02
C18:1 *cis*14	0.16	0.12	0.10	0.11	0.004
C18:1 *cis*15	0.05	0.05	0.03	0.08	0.006
C18:1 *trans*5	0.01	0.01	0.03	0.18	0.01
C18:1 *trans*9	0.19	0.15	0.17	0.49	0.13
C18:1 *trans*10	0.56	0.89	0.48	0.1	0.09
C18:1 *trans*11	0.48 ^b^	0.74 ^a^	0.49 ^b^	0.04	0.01
C18:1 *trans*12	0.13	0.11	0.10	0.27	0.73
Ʃ *trans* C18:1	1.77	1.73	1.38	0.35	0.12
**PUFA**					
C18:2 n-6	4.48 ^b^	6.13 ^a^	4.69 ^b^	0.01	0.52
C18:2 *trans*10. *cis*12 CLA	0.04	0.05	0.05	0.40	0.003
C18:2 *cis*7. *cis*9 CLA	0.01	0.01	0.01	0.92	0.002
C18:2 *cis* 9. *trans*11 CLA	0.32	0.22	0.24	0.26	0.03
C18:2 *trans*9. *cis*11 CLA	0.05	0.07	0.07	0.44	0.01
C18:2 *cis*1. *trans*1 CLA	0.01	0.01	0.01	0.93	0.00
C18:3 n-3	0.51	0.53	0.44	0.22	0.01
C18:3 n-6	0.05	0.06	0.06	0.53	0.03
C20:2 n-6	0.05	0.06	0.05	0.35	0.003
C20:3 n-9	0.32	0.35	0.33	0.64	0.01
C20:3 n-6	0.16 ^b^	0.24 ^a^	0.18 ^b^	0.05	0.01
C20:4 n-6 ARA	1.67 ^b^	2.21 ^a^	1.75 ^b^	0.04	0.03
C20:5 n3 EPA	0.17	0.18	0.22	0.53	0.17
C22:4 n-6	0.12	0.13	0.13	0.88	0.02
C22:5 n-3 DPA	0.36	0.37	0.40	0.94	0.01
C22:6 n-3 DHA	0.12	0.12	0.14	0.82	0.01

C: Control group receiving oat hay and concentrate; Myrt-H: group receiving pellets containing 87% myrtle residues (MR); Myrt-C: group receiving pellets containing 30% MR; SFA: saturated fatty acids; MUFA: monounsaturated fatty acids; PUFA: polyunsaturated fatty acids; a. b.: different letters within the same row (different diets) differ significantly (*p* < 0.05).

**Table 4:** Fatty acid groups (% of total FAME) and ratios of meat from ewes fed myrtle residues.

Item	C	Myrt-H	Myrt-C	*p*	SEM
**⅀SFA**	50.91	51.17	52.24	0.4	0.41
**⅀*cis*_MUFA**	37.83	35.45	36.54	0.44	0.69
**⅀*trans*_MUFA**	2.02	1.93	1.58	0.37	0.13
**⅀MUFA**	39.85	37.38	38.12	0.49	0.73
**⅀CLA**	0.46	0.39	0.41	0.56	0.02
**BCFA**	1.99	1.80	1.82	0.23	0.06
**⅀PUFA**	9.25	11.46	9.64	0.36	0.68
**⅀PUFA n-6**	5.19	8.87	6.04	0.06	1.11
**⅀PUFA n-3**	1.16	1.21	1.20	0.96	0.02
**18:1 10t/C18:1 11t**	1.97 ^b^	0.78 ^a^	1.14 ^b^	0.04	0.15
**DFA**	64.79	65.36	63.57	0.08	0.59
**PUFA/SFA**	0.18	0.22	0.19	0.33	0.01
**n-6/n-3**	5.63 ^b^	7.20 ^a^	5.85 ^b^	0.03	0.49
**AI**	0.69	0.73	0.76	0.27	0.02
**TI**	1.61	1.61	1.69	0.57	0.03

C: Control group receiving oat hay and concentrate; Myrt-H: group receiving pellets containing 87% myrtle residues (MR); Myrt-C: group receiving pellets containing 30% MR; SFA: saturated fatty acids; MUFA: monounsaturated fatty acids; PUFA: polyunsaturated fatty acids; CLA: conjugated linoleic acids; DFA: desirable fatty acids; TI: thrombogenicity index; AI: atherogenicity index. a. b.: different letters within the same row differ significantly (*p* < 0.05).

**Table 5:** Feedstuffs’ antioxidant potential, fatty acids profile (% of total FAME), and chemical composition.

	Ingredients				
Antioxidant and Radical Scavenging Activities	Hay	Concentrate	MR	M-Hay	M-Conc
TPC (mg TAE/g)	6.88	3.07	153.43	72.4	25.17
DPPH radical scavenging activity (mg TE/g)	17.8	17.78	670.47	242.24	127.16
ABTS radical scavenging ability (mg TE/g)	16.2	4.0	406.00	305.53	194.27
**Fatty Acids *(%)***					
C18:0	17.00	8.14	11.52	11.07	9.43
C18:2-n6	16.64	45.19	34.27	31.59	40.98
C18:3-n3	6.33	3.20	4.75	7.05	4.72
SFA	58.44	35.70	44.11	40.92	38.19
MUFA	18.58	15.91	16.87	20.44	16.11
PUFA	22.98	48.39	39.02	38.64	45.70
n6	16.64	45.19	34.27	31.59	40.98
n3	6.33	3.20	4.75	7.05	4.72
n6/n3	16.64	45.19	34.27	31.59	40.98
**Nutritional Composition**					
Dry matter	90.1	92.6	91.8	93	92.1
Crude protein	5.1	12.9	-	10.6	14.9
Total ash	6.2	2.6	-	5	8.5
NDF	63.8	44.98	33.56	37.59	30.85
ADF	36.39	10.21	24.11	20.69	11.29
ADL	3.49	1.25	8.78	5.99	2.91
Tannins (mg TAE/g)	6.88	3.07	153.43	72.41	25.17

MR: distillate myrtle leaves; M-Hay: pellets containing 87% of myrtle residues (MR); M-Conc: pellets containing 30% of MR.
